# Using meta-learning to recommend an appropriate time-series forecasting model

**DOI:** 10.1186/s12889-023-17627-y

**Published:** 2024-01-10

**Authors:** Nasrin Talkhi, Narges Akhavan Fatemi, Mehdi Jabbari Nooghabi, Ehsan Soltani, Azadeh Jabbari Nooghabi

**Affiliations:** 1 Department of Biostatistics, School of Health, Mashhad University of Medical Sciences, Mashhad, Iran; 2 Department of Statistics, Ferdowsi University of Mashhad, Mashhad, Iran; 3 Surgical Oncology Research Center, Mashhad University of Medical Sciences, Mashhad, Iran

**Keywords:** ARIMA, COVID-19, Forecasting, Machine-learning, Meta-learning, TBATS

## Abstract

**Background:**

There are various forecasting algorithms available for univariate time series, ranging from simple to sophisticated and computational. In practice, selecting the most appropriate algorithm can be difficult, because there are too many algorithms. Although expert knowledge is required to make an informed decision, sometimes it is not feasible due to the lack of such resources as time, money, and manpower.

**Methods:**

In this study, we used coronavirus disease 2019 (COVID-19) data, including the absolute numbers of confirmed, death and recovered cases per day in 187 countries from February 20, 2020, to May 25, 2021. Two popular forecasting models, including Auto-Regressive Integrated Moving Average (ARIMA) and exponential smoothing state-space model with Trigonometric seasonality, Box-Cox transformation, ARMA errors, Trend, and Seasonal components (TBATS) were used to forecast the data. Moreover, the data were evaluated by the root mean squared error (RMSE), mean absolute error (MAE), mean absolute percentage error (MAPE), and symmetric mean absolute percentage error (SMAPE) criteria to label time series. The various characteristics of each time series based on the univariate time series structure were extracted as meta-features. After that, three machine-learning classification algorithms, including support vector machine (SVM), decision tree (DT), random forest (RF), and artificial neural network (ANN) were used as meta-learners to recommend an appropriate forecasting model.

**Results:**

The finding of the study showed that the DT model had a better performance in the classification of time series. The accuracy of DT in the training and testing phases was 87.50% and 82.50%, respectively. The sensitivity of the DT algorithm in the training phase was 86.58% and its specificity was 88.46%. Moreover, the sensitivity and specificity of the DT algorithm in the testing phase were 73.33% and 88%, respectively.

**Conclusion:**

In general, the meta-learning approach was able to predict the appropriate forecasting model (ARIMA and TBATS) based on some time series features. Considering some characteristics of the desired COVID-19 time series, the ARIMA or TBATS forecasting model might be recommended to forecast the death, confirmed, and recovered trend cases of COVID-19 by the DT model.

## Introduction

In December 2019, a novel coronavirus emerged in Wuhan City, Hubei province of China [1]. Its high prevalence caused the virus to spread rapidly around the world and became a pandemic. Reports from the World Health Organization (WHO) showed that the virus expanded to all countries of the world, negatively affecting personal life, economy, industry, etc. [2].

This virus could survive on the surface for a few days and transmit rapidly from human to human [3]. The symptoms of this disease were fatigue, general weakness, difficulty breathing, chest pain, sore throat, fever, acute respiratory distress, muscular pain, etc. However, the majority of people had no symptoms [3, 4].

Governments implemented interventions and strategies such as maintaining social distancing, wearing masks, staying at home, not gathering in public places, etc., to reduce the pandemic trend [5]. However, after more than a year and many interventions to deal with the virus, this disease was still the cause of death of many people [6]. Considering its widespread distribution, the virus could recombine the genomes and create a new mutation. Therefore, this infectious disease was likely to appear periodically in humans [1].

If we compare SARS-COV-2 coronavirus with some other previous pandemics, we can observe that SARS-COV-2 had a considerably bigger impact than SARS coronavirus pandemic. In terms of mortality, COVID-19 is comparable with previous flu pandemics. But COVID-19 compared to the swine flu pandemic -also H1N1 (2009) Spanish flu (1918)- seemed relatively severe, because COVID-19 required more people to get hospitalized, while the swine flu pandemic did not [7, 8]. In 2014, Ebola emerged as a virus with an average fatality rate of 50%.

One major difference between Ebola and COVID-19 is the method of spread. Ebola is spread during the last stage of the disease through blood and sweat. Coronavirus is having airborne transmission.

In conclusion, regardless of the mortality rate or the number of confirmed cases, COVID-19 had devastating worldwide impact. Undoubtedly, scientists, statisticians, etc. will continue to learn more about how COVID-19 stacks up against other viruses [9].

In the previous studies, statistical, machine-learning forecasting methods also were applied to forecast these pandemics [10–12].

Forecasting of confirmed cases, death, and recovery in the future informs the increasing or decreasing trend of the COVID-19 disease in the future and makes necessary measures to save people’s lives to be thought of, therefore forecasting models can be very important and helpful.

In the current situation, data analysts have an important role to play. Forecasting the future behavior of the viral infection such as coronavirus with the help of statistical, mathematical, and machine learning models can provide prior useful information to governments and politicians regarding the behavior of the virus and predict the number of infected and death cases in the coming days. Nowadays, statistical techniques and machine learning algorithms are widely used in the medical field with successful results [13].

Time series forecasting has been a very active area of research since the 1950s [14]. The guidelines for time series analysis, finding an appropriate Auto Regressive Integrated Moving Average (ARIMA) model, and investigating autocorrelation function (ACF) and partial autocorrelation function (PACF) values of a time series are summarized in [15]. In the 1990s, the characteristics extracted from univariate time series were used to select the appropriate forecasting model for the first time [14].

Meta-learning supports data mining tasks [16]. The term ‘meta-learning’ was used for the first time in the literature of time series [17]. In the time series area, meta-learning demonstrates the process of automatically acquiring knowledge to identify the best forecasting model, which is based on the machine-learning community [14]. In other words, meta-learning refers to the process of investigating the relationship between learning strategies and tasks [16]. In fact, the main property of meta-learning is to understand the nature of data and learn based on the characteristics extracted from time series, to choose the best forecasting model for a particular data type [16].

**Fig. 1:**
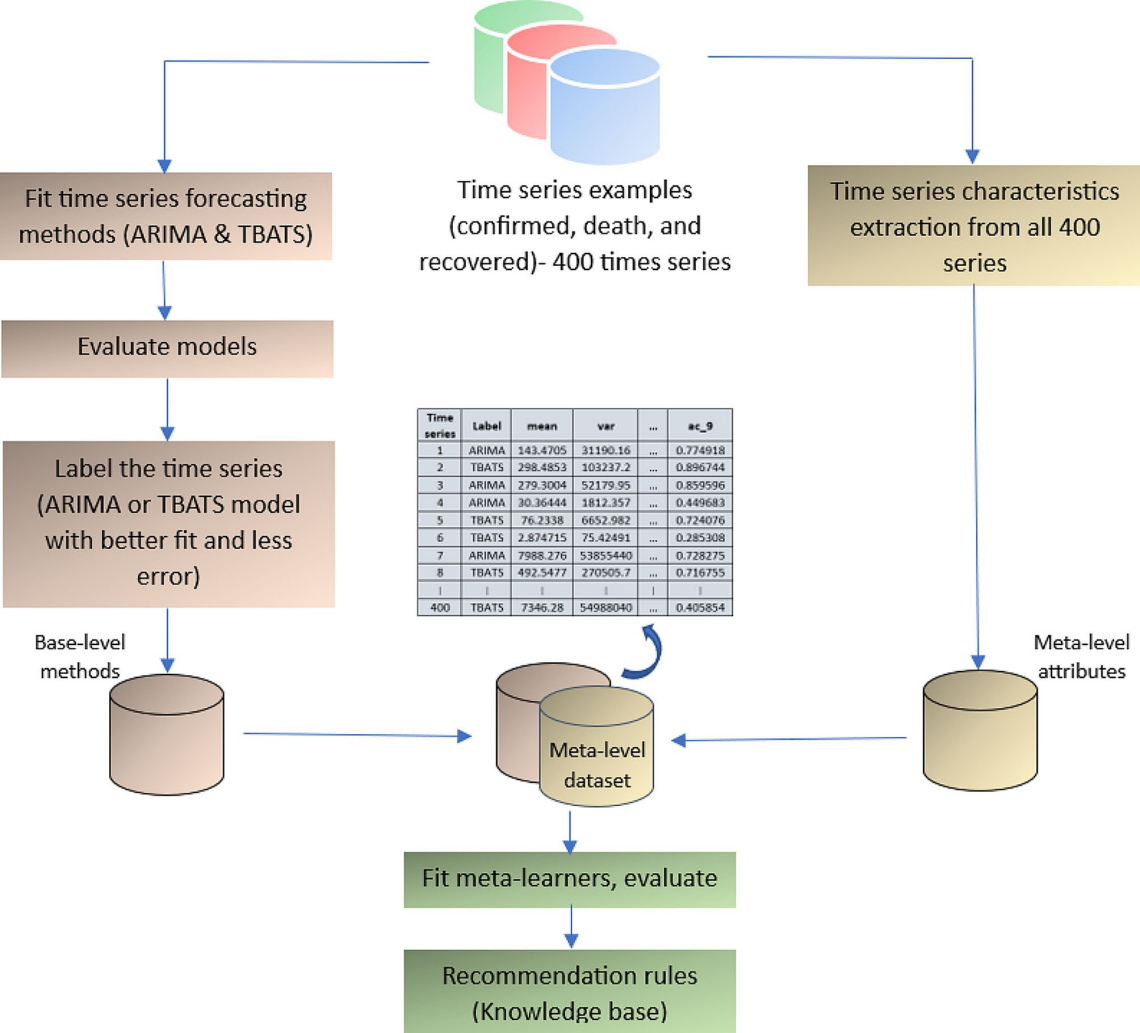
The designed framework of the meta-learning process

The meta-learning framework includes three major steps as follows: (a) fitting the forecasting models and their performance evaluation, (b) extracting the characteristics of time series, and (c) rule induction (Fig. 1), which can lead to a recommender system.

Many studies have been done in this area. For instance, Malki et al. conducted three studies in the field of COVID-19 disease [18–20]. In one of these studies, the Seasonal AutoRegressive Integrated Moving Average (SARIMA) model was applied to predict the spread of the coronavirus in several countries. In another, machine learning approaches are considered to predict the spread of COVID-19 in many countries. In a study conducted by Harbola et al., the COVID-19 outbreak was forecasted using long short-term memory (LSTM). LSTM model showed the trend of infected cases of COVID-19 increased exponentially every week [21].

Therefore, along with all the studies that have been done [22–27], the existence of a recommender system that suggests the appropriate model for making future predictions can also be helpful and practical as well as save time and costs.

In this study, despite spending time and cost, the main goal was to achieve a recommender system design using a meta-learning approach. This system selects and recommends the best forecasting model from two popular forecasting models, Autoregressive Integrated Moving Average (ARIMA) and exponential smoothing state-space model with Trigonometric seasonality, Box-Cox transformation, ARMA errors, Trend, and Seasonal components (TBATS), using time series features. The result of this research can be used for the prevalence of other infectious diseases such as respiratory diseases, etc.

## Methodology

### Data description

In this study, the COVID-19 data included the absolute numbers of confirmed, death, and recovered cases per day from February 20, 2020, to May 25, 2021, for 187 countries. This data was obtained from the GitHub online repository. Therefore, there were a total of 561 series (187 series related to confirmed cases, 187 to death cases, and 187 to recovered cases). We selected a total of 400 series to construct the model, randomly.

### Forecasting methods

ARIMA model was applied to non-stationary time series models and became stationary with operators such as difference, logarithm, root, etc. It is the most well-known model used for time series forecasting problems [25, 27]. The model is a combination of an auto-regressive (AR) model and a moving average (MA) model, and a white noise process [28]. In fact, the ARIMA model is an ARMA time series model that has been differentiated d times and it is indicated by the ARIMA (p,d,q) symbol [29]. Multiplicative seasonal ARIMA models are defined with non-seasonal orders p, d, and q, seasonal orders P, D, and Q, and seasonal period s (ARIMA(p, d, q)(P, D, Q)[s]) [29].

BATS and TBATS are two interesting models of time series able to capture seasonality patterns in modeling series [30]. In fact, the BATS model is an extension of traditional seasonal models or state-space models. In addition, these models handle nonlinearity models using Box-Cox transformation. The B notation in BATS refers to Box-Cox transformation. Other notations (e.g., A, T, and S) in BATS refer to errors of ARIMA, trend, and seasonal components, respectively. A flexible approach of BATS was introduced as TBATS. This model used the Fourier series to the representation of seasonal components of time series [31].

In this study, 80% of the observations at the beginning of each series were used as training data, and the remaining 20% as testing data. Two models, including the ARIMA and TBATS, were fitted to each of the 400 series. Using forecasting evaluation metrics or error measures, time series were labeled based on the most appropriate model among ARIMA and TBATS.

### Error measures

Four error measures were used to evaluate and validate the forecasting performance of the models. All four evaluation metrics measure the difference between the prediction values and the real outcome values or errors. The root mean square error (RMSE), mean absolute error (MAE), mean absolute percentage error (MAPE), and symmetric mean absolute percentage error (SMAPE) were applied to compare the accuracy of forecasting models. Smaller values of these error measures indicate more accurate model prediction. The formulas for these error measures are as follows:

$$ \text{R}\text{M}\text{S}\text{E}=\sqrt{\frac{1}{\text{N}}\sum _{\text{i}=1}^{\text{N}}{({\text{Y}}_{\text{i}}-{\widehat{\text{Y}}}_{\text{i}})}^{2} }$$

$$ \text{M}\text{A}\text{E}=\frac{1}{\text{N}}\sum _{\text{i}=1}^{\text{N}}\left|{\text{y}}_{\text{i}}-{\widehat{\text{y}}}_{\text{i}}\right|,$$

$$ \text{M}\text{A}\text{P}\text{E}=\frac{1}{\text{N}}\sum _{\text{i}=1}^{\text{N}}\frac{|{\text{y}}_{\text{i}}-{\widehat{\text{y}}}_{\text{i}}|}{{\text{y}}_{\text{i}}}\text{*}100\text{\%}$$,

$$ \text{s}\text{M}\text{A}\text{P}\text{E}=\frac{1}{\text{N}}\sum _{\text{i}=1}^{\text{N}}\frac{|{\text{y}}_{\text{i}}-{\widehat{\text{y}}}_{\text{i}}|}{|{\text{y}}_{\text{i}}+{\widehat{\text{y}}}_{\text{i}}|}\text{*}100\text{\%}$$.

### Meta-feature extraction

The various characteristics of time series based on univariate time series structure were investigated [14, 32, 33]. We considered a set of hand-selected features in our study. These features describe the characteristics of time series. The functions available in R software v.4.0.2 were implemented to extract the time series characteristics or meta-features. To summarize the time series structure, 30 characteristics were selected and listed in Table 1.

**Table 1:** Hand-selected extracted features on time series

Number	Feature	Description
1	mean	Mean of series
2	var	Variance of series
3	skewness	Skewness of series
4	kurtosis	Kurtosis of series
5	x_acf1	Sum of the squared of first ACF values of the series
6	x_acf10	Sum of the squared first 10 ACF values of the series
7	diff1_acf1	First ACF value of the differenced series
8	diff1_acf10	Sum of the squared first 10 values of the first-differenced series
9	diff2_acf1	First ACF value of twice differenced series
10	diff2_acf10	Sum of the squared first 10 ACF values of the series
11	ARCH.LM	ARCH LM statistic
12	crossing_points	Number of times the time series crosses the median
13	entropy	Spectral entropy
14	flat_spots	Number of flat spots, calculated by discretizing the series
15	hurst	Hurst exponent
16	lumpiness	Lumpiness
17	nonlinearity	Nonlinearity
18	x_pacf5	Sum of the squared first 5 PACF values of series
19	diff1x_pacf5	Sum of the squared first 5 PACF values of differenced series
20	diff2x_pacf5	Sum of the squared first 5 PACF values of twice-differenced
21	stability	Stability
22	trend	Strength of trend
23	spike	Spikiness
24	linearity	Linearity
25	curvature	Curvature
26	e_acf1	First ACF value of remainder series
27	e_acf10	Sum of the squared first 10 ACF values of remainder series
28	unitroot_kpss	Test statistic based on KPSS test
29	unitroot_pp	Test statistic based on Phillips-Perron test
30	ac_9	Autocorrelation at lag 9

### Meta-learning

The goal of meta-learning is to “…understand how learning itself can become flexible according to the domain or task under study” [34]. The process of meta-learning transforms the problem space into a feature space. Moreover, the extracted meta-features are applied as input and class labels are applied as the outcome in meta-learners. The class labels are the best forecasting algorithm for each time series [35].

The meta-learner may be a machine-learning algorithm. So, there are several supervised machine-learning algorithms [36]. In this study, four machine-learning algorithms, including decision tree (DT), support vector machines (SVM), artificial neural networks (ANN), and random forest (RF) were applied as meta-learners.

DT is a machine-learning and non-parametric method. It is a popular and tree-based method applied to both classification and regression [33, 37]. In fact, the advantage of tree-based methods is that they are flexible and are used to solve non-linear problems with large dimensions and simplify the interpretability of the model [37]. SVM is a powerful and effective technique [38]. It is a learning system from data applied for classification and regression problems [3]. One of the other well-known algorithms in the artificial intelligence area is the ANN algorithm. This algorithm works based on biological human neurons [39]. In many fields, the ANNs have achieved great success in solving various real-world problems. Moreover, these algorithms are recognized as a powerful tool in identifying and exploring the relationship between network inputs (extracted meta-features in the current study) and outputs (created labels in the current study) [39]. The random forest model is one of the most successful ensemble methods [40]. This method is a stronger learner machine than a decision tree learner machine [41].

In the following, to perform this study, the various characteristics of each time series were extracted and the dataset for classification analysis was obtained. Then, this data was randomly divided into training (80%) and testing data (20%). Next, the desired meta-learners were used to predict the ARIMA or TBATS model as a recommended forecasting model for each time series. As well, DT can provide some practical rules for prediction tasks.

## Results

**Fig. 2:**
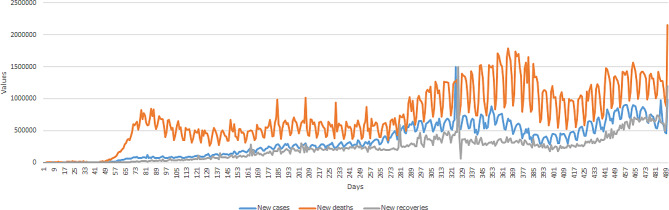
The global trend of confirmed, death, and recovered cases

According to the results (Fig. 2), an increasing and periodic trend was observed in the global trend of confirmed, death, and recovered cases from February 20, 2020, to May 25, 2021. The maximum numbers of infected, death, and recovered cases in the world were 1,498,213, 21,577, and 6,606,167, respectively. For a better representation, the observation value of 6,606,167 was multiplied by 0.01.

Two of the most powerful forecasting models, including ARIMA and TBATS, were fitted on all of the time series. The RMSE, MAE, MAPE, and SMAPE were used to evaluate and find the more appropriate models among ARIMA and TBATS for each time series. Therefore, all the 400 series were labeled with ARIMA or TBATS labels.

In the next phase, we extracted 30 features as meta-features from the available time series. Finding appropriate time series features for classification is not straightforward, as time series analysis is a complex issue. Thus, the used features were hand-selected manually. These features and their descriptions are summarized in Table 1.

After providing the data frame required for the classification task, in the next phase, we intended to classify time series. For this purpose, SVM, DT, ANN, and RF were applied as meta-learners or classifiers.

The 10-fold cross-validation (k-fold CV) method was considered for hyper-parameters tuning and model evaluation on the training and test datasets using RMSE, MAE, MAPE, and SMAPE criteria. Then, the model with the less predicted error was selected. The accuracy of meta-learners is presented in Table 2.

**Table 2:** Accuracy of classification algorithms

Meta-Learner	Train Phase	Test Phase
SVM	0.801	0.786
DT	**0.875**	**0.825**
ANN	0.798	0.775
RF	0.876	0.821

The DT model had a better performance in the classification of time series. The detailed results of the DT classifier, including confusion matrix, accuracy, sensitivity, specificity, etc. are shown in Table 3.

**Table 3:** Confusion matrix of DT algorithm in the train and test phases

(a) Training			(b) Testing		
**Prediction**	**Reference**		**Prediction**	**Reference**	
	**ARIMA**	**TBATS**		**ARIMA**	**TBATS**
**ARIMA**	137	18	**ARIMA**	22	6
**TBATS**	22	143	**TBATS**	8	44
Sensitivity = 86.66%		AUC = 88.73%	Sensitivity = 73.33%		AUC = 78.86%
Specificity = 88.38%		Accuracy = 87.50%	Specificity = 88.00%		Accuracy = 82.50%

**Fig. 3:**
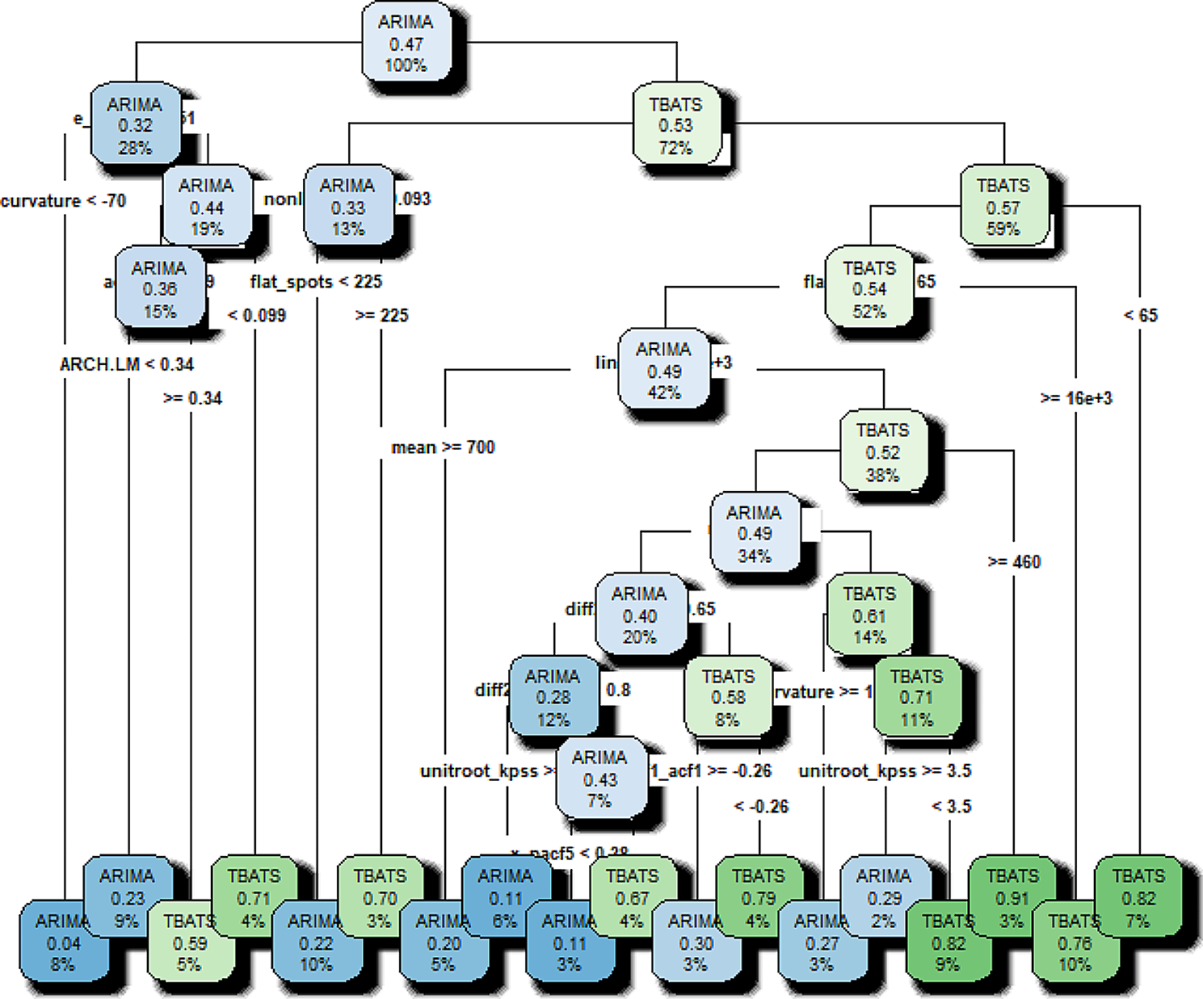
Representation of decision tree

The tree plot is visualized in Fig. 3. The extracted 18 rules are reported in Table 4. Rule 1 shows that if a time series has features such as e_acf1< -0.0513 and curvature< -70.3295, with a 0.962 probability, ARIMA is an appropriate model for forecasting its future trend. In other words, having the values of characteristics e_acf1< -0.0513 and curvature< -70.3295 of a time series, with a 0.962 probability, the appropriate forecasting model will be ARIMA. Meanwhile, in a desired time series, if e_acf1>= -0.0513, nonlinearity > = 0.0931, flat_spots > = 65, linearity < 16070.36, mean < 699.7255, and mean > = 459.6322, then the predicted class would be TBATS with a probability of 0.909. Therefore, using these characteristics, we can predict TBATS as an appropriate forecasting model for this time series.

**Table 4:** Extracted rules of DT algorithm

Rule	Description of rules		Predicted class	Prob
1	If e_acf1< -0.0513 & curvature< -70.3295	Then class is:	ARIMA	0.962
2	If e_acf1< -0.0513 & curvature>= -70.3295 & ac_9 > = 0.098 & ARCH.LM < 0.3356	Then class is:	ARIMA	0.766
3	If e_acf1< -0.0513 & curvature> -70.3295 & ac_9 > = 0.098 & ARCH.LM > = 0.3356	Then class is:	TBATS	0.588
4	If e_acf1< -0.0513 & curvature> -70.3295 & ac_9 < 0.098	Then class is:	TBATS	0.714
5	If e_acf1>= -0.0513 & nonlinearity < 0.0931 & flat_spots < 224.5	Then class is:	ARIMA	0.781
6	If e_acf1>= -0.0513 & nonlinearity < 0.0931 & flat_spots > = 224.5	Then class is:	TBATS	0.700
7	If e_acf1>= -0.0513 & nonlinearity > = 0.0931 & flat_spots > = 65 & linearity < 16070.36 & mean > = 699.7255	Then class is:	ARIMA	0.800
8	If e_acf1>= -0.0513 & nonlinearity > = 0.0931 & flat_spots > = 65 & linearity < 16070.36 & mean < 699.7255 & mean < 459.6322 & diff2_acf1>=-0.6465 & diff2x_pacf5 > = 0.7956 & unitroot_kpss > = 1.0595	Then class is:	ARIMA	0.888
9	If e_acf1=> -0.0513 & nonlinearity > = 0.0931 & flat_spots > = 65 & linearity < 16070.36 & mean < 699.7255 & mean < 459.6322 & diff2_acf1>=-0.6465 & diff2x_pacf5 > = 0.7956 & unitroot_kpss < 1.0595 & x_pacf5 < 0.3818	Then class is:	ARIMA	0.888
10	If e_acf1=> -0.0513 & nonlinearity > = 0.0931 & flat_spots > = 65 & linearity < 16070.36 & mean < 699.7255 & mean < 459.6322 & diff2_acf1>=-0.6465 & diff2x_pacf5 > = 0.7956 & unitroot_kpss < 1.0595 & x_pacf5 > = 0.3818	Then class is:	TBATS	0.666
11	If e_acf1>= -0.0513 & nonlinearity > = 0.0931 & flat_spots > = 65 & linearity < 16070.36 & mean < 699.7255 & mean < 459.6322 & diff2_acf1>=-0.6465 & diff2x_pacf5 < 0.7956 & diff1_acf1>=-0.2608	Then class is:	ARIMA	0.700
12	If e_acf1>= -0.0513 & nonlinearity > = 0.0931 & flat_spots > = 65 & linearity < 16070.36 & mean < 699.7255 & mean < 459.6322 & diff2_acf1>=-0.6465 & diff2x_pacf5 < 0.7956 & diff1_acf1<-0.2608	Then class is:	TBATS	0.785
13	If e_acf1>= -0.0513 & nonlinearity > = 0.0931 & flat_spots > = 65 & linearity < 16070.36 & mean < 699.7255 & mean < 459.6322 & diff2_acf1<-0.6465 & curvature > = 101.7799	Then class is:	ARIMA	0.727
14	If e_acf1>= -0.0513 & nonlinearity > = 0.0931 & flat_spots > = 65 & linearity < 16070.36 & mean < 699.7255 & mean < 459.6322 & diff2_acf1<-0.6465 & curvature < 101.7799 & unitroot_kpss > = 3.4560	Then class is:	ARIMA	0.714
15	If e_acf1>= -0.0513 & nonlinearity > = 0.0931 & flat_spots > = 65 & linearity < 16070.36 & mean < 699.7255 & mean < 459.6322 & diff2_acf1<-0.6465 & curvature < 101.7799 & unitroot_kpss < 3.4560	Then class is:	TBATS	0.821
16	If e_acf1>= -0.0513 & nonlinearity > = 0.0931 & flat_spots > = 65 & linearity < 16070.36 & mean < 699.7255 & mean > = 459.6322	Then class is:	TBATS	0.909
17	If e_acf1>= -0.0513 & nonlinearity > = 0.0931 & flat_spots > = 65 & linearity > = 16070.36	Then class is:	TBATS	0.757
18	If e_acf1>= -0.0513 & nonlinearity > = 0.0931 & flat_spots > = 65	Then class is:	TBATS	0.818

Prob is abbreviated Probability

## Discussion

Today, forecasting is widely applied in many fields, such as marketing, finance, healthcare, etc. An accurate forecast of the future can be very helpful and provide information on efficiency and cost reduction [42]. Machine learning has grown rapidly and dramatically in the fields of medicine and healthcare [43]. Moreover, it has been used in the field of prediction with successful results [43].

Also, it is a modern method including sophisticated algorithms used in time series and forecasting. In fact, machine learning attempts to discover and extract the patterns and concepts embedded in large data and predict the desired target [44].

In this study, to recommend ARIMA and TBATS forecasting models, the meta-learner of the meta-learning process (DT algorithm) achieved an accuracy of 87.50% and 82.50% in the training and test phases, respectively. Two other meta-learners (i.e., SVM and ANN algorithms) had less accuracy than the DT algorithm in both the training and test phases.

The sensitivity and specificity in the training phase of the DT algorithm were obtained as 86.66% and 88.38%, respectively. In addition, these values in the test phase were 73.33% and 88%, respectively. Thus, the meta-learning approach can predict the appropriate forecasting model (ARIMA and TBATS) with 82.50% accuracy.

It should also be noted that initially four of the strongest statistical models for time series forecasting, including ARIMA, TBATS, ETS (Error Trend and Seasonality, or exponential smoothing), and multiple aggregation prediction algorithm (MAPA) were considered. In the labeling phase, ETS and MAPA had a low frequency and were excluded. Thus, the analyses were performed using the ARIMA and TBATS models.

To the best of our knowledge, this approach has not been applied to recommend a forecasting model based on meta-learning so far. However, many studies in different countries have been conducted to find the best forecasting model with the least forecasting error.

In our previous study [22], the appropriate models to forecast the number of confirmed and death cases were identified the MLP and Holt-Winter model. The web application for visualizing the results is available at.

http://shiny.um.ac.ir/jabbarinm/Covid19/.

Some previous studies have concluded that machine-learning models performed better than classical models such as the ARIMA. Yadav et al. applied some models such as the support vector regression (SVR) model to forecast the future number of total, active, and recovered cases. They also compared the results of the proposed method with other well-known regression models such as simple linear regression and polynomial regression [3].

Yang et al. (2020) used the ARIMA models to forecast the number of new confirmed and death cases in Italy [45]. The ANN was applied by Farooq and Bazaz in the five worst-affected states of India. An online incremental learning technique was performed along with the ANN model. They forecasted the future behavior of COVID-19 disease for the coming 30 days [46].

Christie et al. compared three forecasting methods, including ARIMA, single exponential smoothing (SES), and double exponential smoothing (DES) using the MAPE, and RMSE measures. They showed that the ARIMA is the best model for forecasting COVID-19 disease [47].

Rostami-Tabar and Rendon-Sanchez used a simple multiple linear regression model using the calls received in a call center (phone call data) and fitted the ARIMA, ETS, seasonal naive, prophet, and a regression model without call data. They concluded that the simple multiple linear regression model with call data performed better than other models [48]. It is believed that the models used in this study are very accurate with important predictive variables and high predictability. However, the main limitations of this study include the unavailability of more data and effective predictor variables.

Moftakhar et al. used two ANN and ARIMA models to forecast the number of future cases in the coming 30 days in Iran. They concluded that the ARIMA model was a more accurate method [24], which is similar to our results.

The MLP model proposed by Pantoh et al. was identified as an appropriate model for forecasting the numbers of confirmed, death, and recovered cases using cumulative data [4].

Khan, Saeed, and Ali used the daily absolute confirmed, death, and recovered cases in Pakistan from March 8 to June 27, 2020. They fitted a VAR model to forecast new infected cases and new recovered cases in the next 10 days, i.e. on the 3rd of July [49].

It should be noted that it cannot be said with certainty that machine-learning models perform better than other existing models or that classical models perform better than machine-learning models. Each model can have different results over a time window compared to other models. This depends on the type and nature of the data, the circumstances, and the time window under consideration.

This study has some limitations. First, data for some countries were not fully reported and thus were not usable and hence, we had to exclude them. Second, even though we considered four of the strongest statistical forecasting models, two models ETS and MAPA were selected as appropriate models for a few time series, and as a result, we had to leave these two models aside and the study process continued with the other two models. Third, machine learning forecasting models were not used along with statistical models due to complexity, time-consuming, and cost, and therefore, only statistical forecasting models were used. Fourth, there are various machine-learning algorithms that can be used as meta-learners, and four of them were compared in the current study due to the extensive and time-consuming work. However, the index values showed that the final model (DT) has relatively accurate predictive ability.

## Conclusion

In this study, we achieved a recommender system to select a forecasting model among ARIMA and TBATS using the meta-learning process. Our results showed that among the four meta-learners, namely SVM, DT, ANN, and RF, the DT algorithm had a better predictive accuracy. Therefore, the DT algorithm with 87.50% accuracy in training and 82.50% accuracy in the test phase as the best model, was provided some practical rules. These rules recommended one of two models ARIMA and TBATS to forecast the health time series data such as the confirmed, death, and recovered COVID-19 cases in each country according to the characteristics of their time series.

## Data Availability

The dataset used to train and evaluate the models is publicly available at https://ourworldindata.org/coronavirus-source-data/. Additionally, datasets used and/or analyzed during the current study are available from the corresponding author on reasonable request.
